# Comparison of epidemiological characteristics and transmissibility of different strains of COVID-19 based on the incidence data of all local outbreaks in China as of March 1, 2022

**DOI:** 10.3389/fpubh.2022.949594

**Published:** 2022-09-15

**Authors:** Yan Niu, Li Luo, Shiting Yang, Guzainuer Abudurusuli, Xiaoye Wang, Zeyu Zhao, Jia Rui, Zhuoyang Li, Bin Deng, Weikang Liu, Zhe Zhang, Kangguo Li, Chan Liu, Peihua Li, Jiefeng Huang, Tianlong Yang, Yao Wang, Tianmu Chen, Qun Li

**Affiliations:** ^1^Public Health Emergency Center, Chinese Center for Disease Control and Prevention, Beijing, China; ^2^State Key Laboratory of Molecular Vaccinology and Molecular Diagnostics, School of Public Health, Xiamen University, Fujian, China; ^3^School of Basic Medical Sciences, Cheeloo College of Medicine, Shandong University, Jinan, China

**Keywords:** SARS-CoV-2, effective reproduction number, variant, transmissibility, Delta variant, Omicron variant

## Abstract

**Background:**

The epidemiological characteristics and transmissibility of Coronavirus Disease 2019 (COVID-19) may undergo changes due to the mutation of Severe Acute Respiratory Syndrome Coronavirus 2 (SARS-CoV-2) strains. The purpose of this study is to compare the differences in the outbreaks of the different strains with regards to aspects such as epidemiological characteristics, transmissibility, and difficulties in prevention and control.

**Methods:**

COVID-19 data from outbreaks of pre-Delta strains, the Delta variant and Omicron variant, were obtained from the Chinese Center for Disease Control and Prevention (CDC). Case data were collected from China's direct-reporting system, and the data concerning outbreaks were collected by on-site epidemiological investigators and collated by the authors of this paper. Indicators such as the effective reproduction number (*R*_eff_), time-dependent reproduction number (*R*_t_), rate of decrease in transmissibility (*RDT*), and duration from the illness onset date to the diagnosed date (*D*_*ID*_)/reported date (*D*_*IR*_) were used to compare differences in transmissibility between pre-Delta strains, Delta variants and Omicron variants. Non-parametric tests (namely the Kruskal-Wallis H and Mean-Whitney U tests) were used to compare differences in epidemiological characteristics and transmissibility between outbreaks of different strains. *P* < 0.05 indicated that the difference was statistically significant.

**Results:**

Mainland China has maintained a “dynamic zero-out strategy” since the first case was reported, and clusters of outbreaks have occurred intermittently. The strains causing outbreaks in mainland China have gone through three stages: the outbreak of pre-Delta strains, the outbreak of the Delta variant, and outbreaks involving the superposition of Delta and Omicron variant strains. Each outbreak of pre-Delta strains went through two stages: a rising stage and a falling stage, Each outbreak of the Delta variant and Omicron variant went through three stages: a rising stage, a platform stage and a falling stage. The maximum *R*_eff_ value of Omicron variant outbreaks was highest (median: 6.7; ranged from 5.3 to 8.0) and the differences were statistically significant. The *RDT* value of outbreaks involving pre-Delta strains was smallest (median: 91.4%; [IQR]: 87.30–94.27%), and the differences were statistically significant. The *D*_*ID*_ and *D*_*IR*_ for all strains was mostly in a range of 0–2 days, with more than 75%. The range of duration for outbreaks of pre-Delta strains was the largest (median: 20 days, ranging from 1 to 61 days), and the differences were statistically significant.

**Conclusion:**

With the evolution of the virus, the transmissibility of the variants has increased. The transmissibility of the Omicron variant is higher than that of both the pre-Delta strains and the Delta variant, and is more difficult to suppress. These findings provide us with get a more clear and precise picture of the transmissibility of the different variants in the real world, in accordance with the findings of previous studies. *R*_eff_ is more suitable than *R*_t_ for assessing the transmissibility of the disease during an epidemic outbreak.

## Introduction

The Corona Virus Disease 2019 (COVID-19) has caused serious strains on the worldwide public health systems since the outbreak in late 2019 (1, 2). Over time, Severe Acute Respiratory Syndrome Coronavirus 2 (SARS-CoV-2) has continued to evolve and mutate, producing a variety of SARS-CoV-2 variants (3–5). The Delta variant (B.1.617.2) was first reported in India in December 2020 and became the main variant in most parts of the world in the second half of 2021. A new Variant of Concern (VOC), the Omicron variant (B.1.1.529), was reported on November 26, 2021 (6), and quickly became the dominant global variant. The continuing mutation of SARS-CoV-2 strains resulted in changes in the epidemiological characteristics and transmissibility of Coronavirus Disease 2019 (COVID-19). Studies have found that the infectivity rate of the Delta variant is 97 or 100% higher than that of pre-Delta strains (7), and the rapid spread of the Omicron variant in Gauteng Province, South Africa confirmed its high infectivity rate (8).

The value of the Reproductive number (*R*) is a useful indicator of the status of an outbreak (9). A value of *R* > 1 reflects active community transmission. The basic reproduction number (*R*_0_) refers to the estimated number of secondary infections caused by an infected individual in a susceptible population of susceptible people during the infectious cycle. This definition assumes a complete lack of immunity in all individuals, absent any external intervention measures, such as isolation, vaccination, etc. (10). *R*_0_ applies to a situation of maximal transmissibility of the disease. When the population is not completely susceptible, or intervention measures are taken, the transmissibility of the disease should be measured by the effective reproduction number (*R*_eff_). In addition, there is an indicator derived from *R*_eff_, namely the time-dependent reproduction number (*R*_t_), which can be regarded as time-varying estimate of *R*_eff_ (11) reflecting the instantaneous transmissibility of the case at a certain point in time.

The Delta variant is characterized by stronger infectivity, higher viral load, a shorter incubation period, and rapid onset. Studies have shown that the *R*_0_ of the Delta variant is about 7.0, absent any intervention (12), which is much higher than the 2.2–3.77 range seen in the early stages of the epidemics (13–15). Data from South Africa indicates that the positivity rate of the Omicron variant increased from 1 to 30% within 2 weeks, and subsequent cases also increased exponentially (8). At present, with regards to the local mainland China outbreaks, there is a lack of comprehensive research utilizing first-hand incidence data, nor is there any comparative analysis of the epidemiological characteristics and transmissibility of the different strains. The purpose of this study is to review the aggregated data concerning all outbreaks in China since the first Wuhan-related wave, using multiple indicators to clarify the differences in epidemiological characteristics and transmissibility of different strains. The research also compares *R*_eff_ and *R*_t_, concluding that *R*_eff_ is more suitable than *R*_t_ in evaluating the transmissibility of the disease. The goal is to develop more effective prevention and control strategies, and provide better assessment of the outbreak trajectories of other variants that may occur in the future.

## Methods

### Data collection

**Figure 1:**
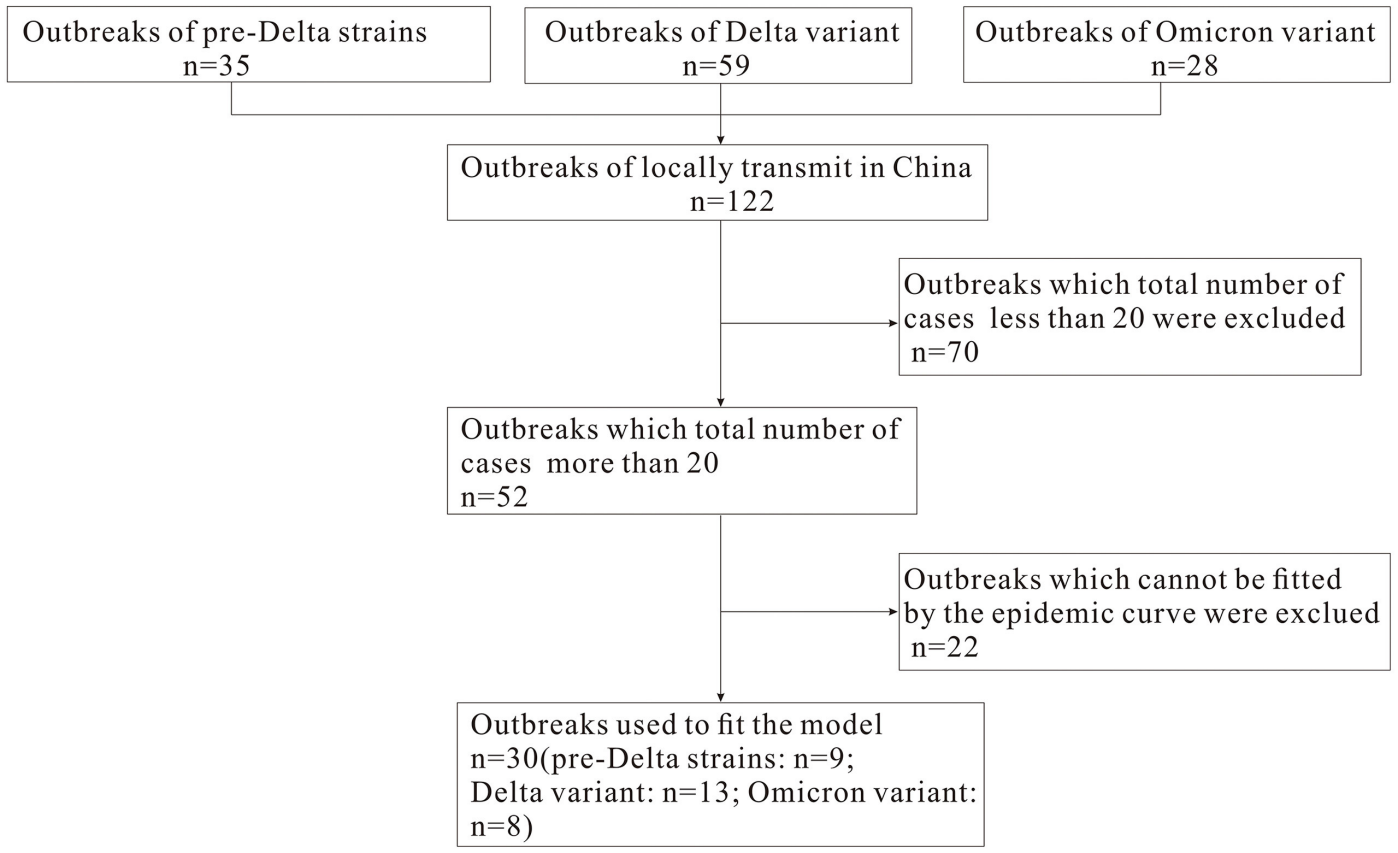
Flow chart of select outbreaks for fitting *R*_eff_ and *R*_t_ value.

In this study, we define an “outbreak” as an event that involving more than one local case, and where the virus strain is confirmed through epidemiological investigation and genome sequencing to be unrelated to previous outbreaks in other regions. The COVID-19 data concerning pre-Delta strains, and Delta and Omicron variants of SARS-CoV-2 from March 28, 2020 to March 1, 2022 were collected to analyze the epidemiological characteristics of COVID-19. Based on epidemic curves and the total number of cases during each outbreak (excluding outbreaks that totaled <20 cases, and which did not fit the epidemic curve), we selected nine pre-Delta strain outbreaks, 13 Delta variant outbreaks and eight Omicron variant outbreaks in order to calculate the *R*_eff_ and *R*_t_ values (Figure 1). Daily incidence data for each outbreak were gathered from the Chinese Center for Disease Control and Prevention (CDC). Case data came from China's direct-reporting system network, and outbreak data was collected by the on-site epidemiological investigators and collated by the authors of this paper. The demographics were collected from the Statistical Yearbook of the locations where the outbreak occurred.

### Estimating the reproductive number (*R*)

#### Estimating the R_eff_

In scenarios where there are interventions during the prediction of outbreak trends, the *R*_eff_ can often replace the *R*_0_ as an indicator of virus transmissibility (16).

The equation is as follows:


$$\begin{array}{l} R_{eff}=\frac{\beta \text{S}}{\gamma } \end{array}$$

The susceptible-exposed-recovered (SEIR) propagation dynamics model was used for the calculation of *R*_eff_. In the SEIR model, the population is divided into four categories: susceptible (*S*), exposed (*E*), infectious (*I*), and recovered (*R*).

The equations of the model are as follows:


$$\begin{array}{lll} \frac{dS}{dt}\; & = & \;-\beta SI\\ \frac{dE}{dt}\; & = & \;\beta SI\;-\;\omega E\\ \frac{dI}{dt}\; & = & \;\omega E\;-\;\gamma I-fI\\ \frac{dR}{dt}\; & = & \;\gamma I \end{array}$$

There are four parameters (β, ω, γ, *f*) in the SEIR model. The parameter values of different strains are shown in Table 1. The total population was set based upon the statistical yearbook data of the location where the outbreak occurred.

**Table 1:** Description and source of parameters.

**Parameter**	**Description**	**Unit**	**Value (SD)**	**Range**	**References**
**Pre-Delta strains**					
ω	Incubation period	/Days	5	2.0–18.0	(17–19)
γ	Infectious period	/Days	5	2.7–8.0	(20–22)
SI	Serial interval	/Days	4.2 (4.0)	4.2–7.5 (0.9–5.8)	(22, 23)
**Delta variant**					
ω	Incubation period	/Days	3	3.0–6.02	(24–26)
γ	Infectious period	/Days	5	5	(26)
SI	Serial interval	/Days	2.3 (3.4)	2.3–3.7 (3.4–5.0)	(27–29)
**Omicron variant**					
ω	Incubation period	/Days	3	3–4.2	(30–32)
γ	Infectious period	/Days	5	–	
SI	Serial interval	/Days	2.8 (1.6)	2.8–3.3 (1.6–3.4)	(30–32)

#### Estimating the R_t_

*R*_t_ and its 95% confidence interval were estimated based on a 5-day moving average using a Bayesian framework based on a time series of the number of new cases of COVID-19 and serial intervals (*SI*) (Table 1) (10). In Supplementary Figures S1–S3, the gray horizontal dotted line indicates *R*_t_ = 1, below which sustained transmission is unlikely as long as intervention measures are maintained.

In this study, we fitted the epidemic curve of the COVID-19 according to the epidemic trend of the disease and the implementation nodes of the prevention and control measures. When comparing the *R*_eff_/*R*_t_ of different strains, we selected the *R*_eff_/*R*_t_ value fitted to the rising stage of each epidemic for analysis, because the *R*_eff_/*R*_t_ at this stage are generally represent the *R*_eff_/*R*_t_ prior to the implementation of various prevention and control measures (except vaccination), which are the closest to *R*_0_, and thus can better restore the transmissibility of the virus itself.

### The rate of decrease in transmissibility

Our study also evaluated the effectiveness of outbreak prevention and control measures in each outbreak through the decline range of *R*_eff_. Prior to any public health and social measures (PHSMs), the epidemic curve will manifest in the natural rising stage. During this time, the *R*_eff_ is denoted as *R*_eff1_. When PHSMs are implemented to control the outbreak situation, the daily number of new cases decreases, and the *R*_eff_ is denoted as *R*_eff2_ during the falling stage. If the outbreak is difficult to control and the epidemic curve enters the rising period followed by the plateau period and then the falling period, the *R*_eff_ of the outbreak status in the plateau period is denoted as $R_{\text{ef}{\text{f}}^{\text{′}}}$. The rate of decrease in transmissibility (*RDT*) is calculated to analyze the decline of transmissibility of each outbreak after effective intervention.


$$\begin{array}{l} \text{RDT}\left(\%\right)\;=\;\frac{\left(R_{eff1}\;-\;R_{eff2}\right)}{R_{eff1}}\;*\;100. \end{array}$$

### Statistics analysis

The calibration between incidence data and the SEIR model was performed using the least squares method. The coefficient of determination (*R*^2^) was used to evaluate the goodness-of-fit. Berkeley Madonna ver. 8.3.18 (developed by Robert Macey and George Oster of the University of California at Berkeley, CA, USA) was used for parameter fitting and model simulation. The “EpiEstim” package in the R software (version 3.6.0, R Core Team, Austria) was used for *R*_t_ estimated. SPSS 21.0 (IBM Corp, Armonk, NY, USA) was used for the goodness-of-fit by calculating the coefficient of determination (*R*^2^). GraphPad Prism 7.0 (GraphPad Software, La Jolla, CA) was used for figure production. Non-parametric tests (namely Kruskal-Wallis H and Mean-Whitney *U*-tests) were used to compare differences in the epidemiological characteristics and transmissibility between outbreaks of pre-Delta strains, the Delta variant and the Omicron variant. We calculated the test statistics (*H* value/*U* value), determined the *P*-value, and then made statistical inference, *P* < 0.05 indicating that the difference was statistically significant.

## Results

### Epidemiological characteristics of COVID-19 transmitted locally in China

**Figure 2:**
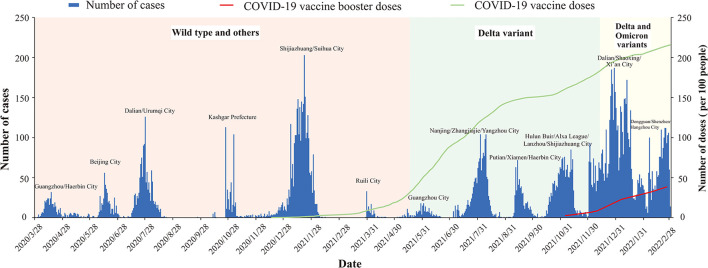
Epidemic curve COVID-19 transmitted locally in China from March 28, 2020 to March 1, 2022.

Mainland China has maintained a “dynamic zero-out strategy” since the first case was reported, and during subsequent intermittent clusters of outbreaks. After epidemiological investigation and genome sequencing, the virus strains involved in each outbreak were confirmed to be unrelated to previous outbreaks. The strains causing the outbreaks in mainland China have manifested in three stages: the outbreak of pre-Delta strains, the outbreak of the Delta variant, and the superposition of outbreaks of Delta and Omicron variant strains. During the outbreak period of each strain, there were peaks in the epidemic curve. On May 21, 2021, Guangzhou City reported the first outbreak of the Delta variant in China, and subsequently Delta variant outbreaks occurred in many cities. On December 15, 2021, Tianjin reported the first outbreak of the Omicron variant in China. Since then, domestic outbreaks have exhibited characteristics of multi-point and frequent occurrence. Outbreaks of Delta and Omicron variants have occurred in superposition, and the number of locally confirmed cases has been at a high level. Doses of COVID-19 vaccine in mainland China have gradually increased (including the booster vaccine) (Figure 2).

In Table 2, the numbers of cases for each strains is a summation of all outbreaks of this strain. Individuals infected with pre-Delta strains, Delta variants and Omicron variants were predominantly farmers, housekeepers and the unemployed, and students, respectively; Individuals infected with pre-Delta strains, Delta variants and Omicron variants were mainly in the age group of 15–44 years of age (Table 2).

**Table 2:** Epidemiological characteristics of COVID-19 transmitted locally in China.

**Variables**	**Pre-Delta strains (5,776)**		**Delta variant (7,660)**		**Omicron variant (905)**	
	**Number of cases**	**Percentage (%)**	**Number of cases**	**Percentage (%)**	**Number of cases**	**Percentage (%)**
**Occupation**						
Nanny	5	0.09	15	0.20	1	0.11
Unknowing	210	3.64	64	0.84	0	0.00
Catering industry	237	4.10	179	2.34	8	0.88
Cadre staff	249	4.31	519	6.78	29	3.20
Worker	376	6.51	1,138	14.86	18	1.99
Service personnel in public places	19	0.33	145	1.89	3	0.33
Seaman and long-distance driver	4	0.07	9	0.12	1	0.11
Housework and unemployment	856	14.82	1,224	**15.98**	175	19.34
Teacher	49	0.85	124	1.62	22	2.43
Retired	336	5.82	534	6.97	13	1.44
Migrant worker	20	0.35	86	1.12	0	0.00
Herdsman	1	0.02	2	0.03	0	0.00
Farmer	1,749	**30.28**	785	10.25	127	14.03
Others	352	6.09	488	6.37	46	5.08
Diaspora children	152	2.63	188	2.45	18	1.99
Business services	406	7.03	749	9.78	11	1.22
Student	615	10.65	1,132	14.78	409	**45.19**
Medical personnel	65	1.13	154	2.01	7	0.77
Childcare	75	1.30	122	1.59	17	1.88
Fishing (boat) people	0	0.00	3	0.04	0	0.00
**Age group**						
≤ 14years old	644	11.15	953	12.44	156	17.24
15–44 years old	2,608	**45.15**	3,535	**46.15**	522	**57.68**
45–64 years old	1,808	31.30	2,256	29.45	168	18.56
≥65 years old	716	12.40	916	11.96	59	6.52
**DID**						
0–2	4,347	**75.26**	6,219	**81.19**	771	**85.29**
3–5	974	16.86	1,163	15.18	115	12.72
6–10	337	5.83	231	3.02	15	1.66
11–20	105	1.82	45	0.59	3	0.33
≥21	13	0.23	2	0.03	0	0.00
**DIR**						
0–2	4,724	**81.79**	6,674	**87.13**	771	**85.29**
3–5	669	11.58	756	9.87	115	12.72
6–10	293	5.07	193	2.52	15	1.66
11–20	86	1.49	35	0.46	3	0.33
≥21	4	0.07	2	0.03	0	0.00

### Curve fitting results

Based on the epidemic curve and total number of cases during each outbreak, we selected outbreaks of nine pre-Delta strains, 13 Delta variant outbreaks and eight Omicron variant outbreaks for model fitting. It can be seen from Supplementary Figures S1–S3 that the model fits well with the outbreak data (*R*^2^ are >0.40 and the *P*-values are <0.05).

As can be seen from Supplementary Figures S1–S3. Each outbreak of the pre-Delta strains went through two stages: a rising stage and a falling stage; each outbreak of Delta and Omicron variants went through three stages: a rising stage, a platform stage and a falling stage. The *R*_eff_ value of each outbreak of pre-Delta strains was below 4, and the *R*_eff_ value of each outbreak of Delta and Omicron variants was above 4. At the beginning of each outbreak, the *R*_eff_ and *R*_t_ values were >1. After strong intervention measures were applied, both the *R*_eff_ and *R*_t_ values would drop lower than 1, suggesting that outbreaks were under control. However, the timing of the values <1 were significantly different in many outbreaks.

### Comparison of transmissibility between different strains

**Figure 3:**
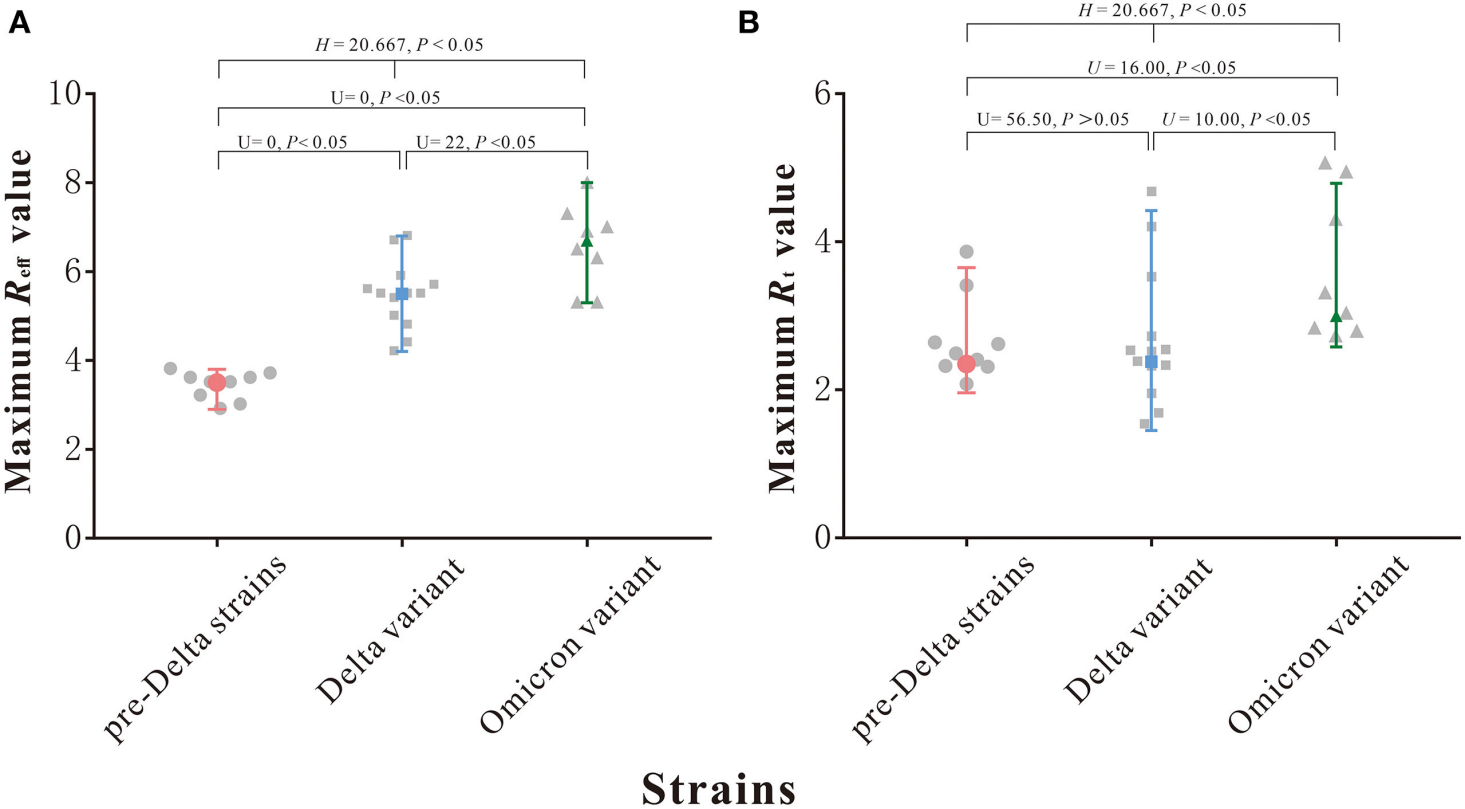
Maximum *R*_eff_ and *R*_t_ value of 9 pre-Delta strains outbreaks and 13 Delta variant outbreaks and 8 Omicron variant outbreaks. **(A)** Maximum *R*_eff_ value. **(B)** Maximum *R*_t_ value.

As can be seen from Figure 3A, the maximum *R*_eff_ values of outbreaks of nine pre-Delta strains ranged from 2.9 to 3.8, with a median of 3.5; the maximum *R*_eff_ values of 13 Delta variant outbreaks ranged from 4.2 to 6.8, with a median of 5.5; and the maximum *R*_eff_ values of eight Omicron variant outbreaks ranged from 5.3 to 8.0, with a median of 6.7. The median of the maximum *R*_eff_ value of Omicron variant outbreaks was highest. The differences between any two groups were statistically significant (*H* = 20.667, *P* < 0.05).

As can be seen from Figure 3B, the maximum *R*_t_ values of outbreaks of nine pre-Delta strains ranged from 1.96 to 3.65, with a median of 2.35; the maximum *R*_t_ values of 13 Delta variant outbreaks ranged from 1.45 to 4.42, with a median of 2.38; and the maximum *R*_t_ values of eight Omicron variant outbreaks ranged from 2.58 to 4.79, with a median of 3.00. The median of the maximum *R*_t_ value of Omicron variant outbreaks was highest, but the differences between three groups were statistically significant (*H* = 8.461, *P* > 0.05), but though the differences between Delta variant and pre-Delta strains was not statistically significant (U = 56.50, *P* > 0.05).

The median of *RDT* of outbreaks involving pre-Delta strains was 91.4% (inter-quartile range [IQR]: 87.30–94.27%); the *RDT* median of Delta variant outbreaks was 100% (inter-quartile range [IQR]: 98.08–100%); and the median of *RDT* of Omicron variant outbreaks was 98.75% (inter-quartile range [IQR]: 96.05–100%). The *RDT* value of outbreaks due to pre-Delta strains was the smallest (Table 3), and the differences between any two groups were statistically significant (*H* = 17.998, *P* < 0.05).

**Table 3:** The RDT of nine pre-Delta strains, 13 Delta variant and eight Omicron variant outbreaks.

**Stains**	**Outbreaks**	**RDT (%)**	**Median (IQR)**
Pre-Delta strains	2020-3-Harbin City	94.59	91.43 (87.30–94.27)
	2020-3-Guangzhou City	91.43	
	2020-6-Fengtai District, Beijing	85.71	
	2020-7-Urumqi City	90.63	
	2020-7-Dalian City	82.76	
	2020-11-Dalian City	92.11	
	2021-1 Shijiazhuang City	96.67	
	2021-1-Beijing City	93.94	
	2021-1-Suihua City	88.89	
Delta variant	2021-5-Guangzhou City	95.83	100 (98.08–100)
	2021-7-Nanjing City	100	
	2021-7-Yangzhou City	98.15	
	2021-7-Zhangjiajie City	98	
	2021-9-Putian and Quanzhou City	100	
	2021-9-Xiamen City	100	
	2021-9-Harbin City	99.3	
	2021-10-Ejina Banner	100	
	2021-10-Heihe City	100	
	2021-10-Shijiazhuang City	100	
	2021-10-Dalian City	100	
	2021-11-Shaoxing City	100	
	2021-11-Xi'an City	96.61	
Omicron variant	2021-12-Tianjin City	100	98.75 (96.05–100)
	2022-1-Anyang City	97.5	
	2022-1-Zhuhai City	94.34	
	2022-1-Hangzhou City	100	
	2022-2-Suzhou City	97.26	
	2022-2-Chengdu City	100	
	2022-2-Dongguan City	95.65	
	2022-1-Shenzhen City	100	

### Duration of the illness from onset date to the diagnosis date (*D_*ID*_*)/reported date (*D_*IR*_*) of outbreaks

**Figure 4:**
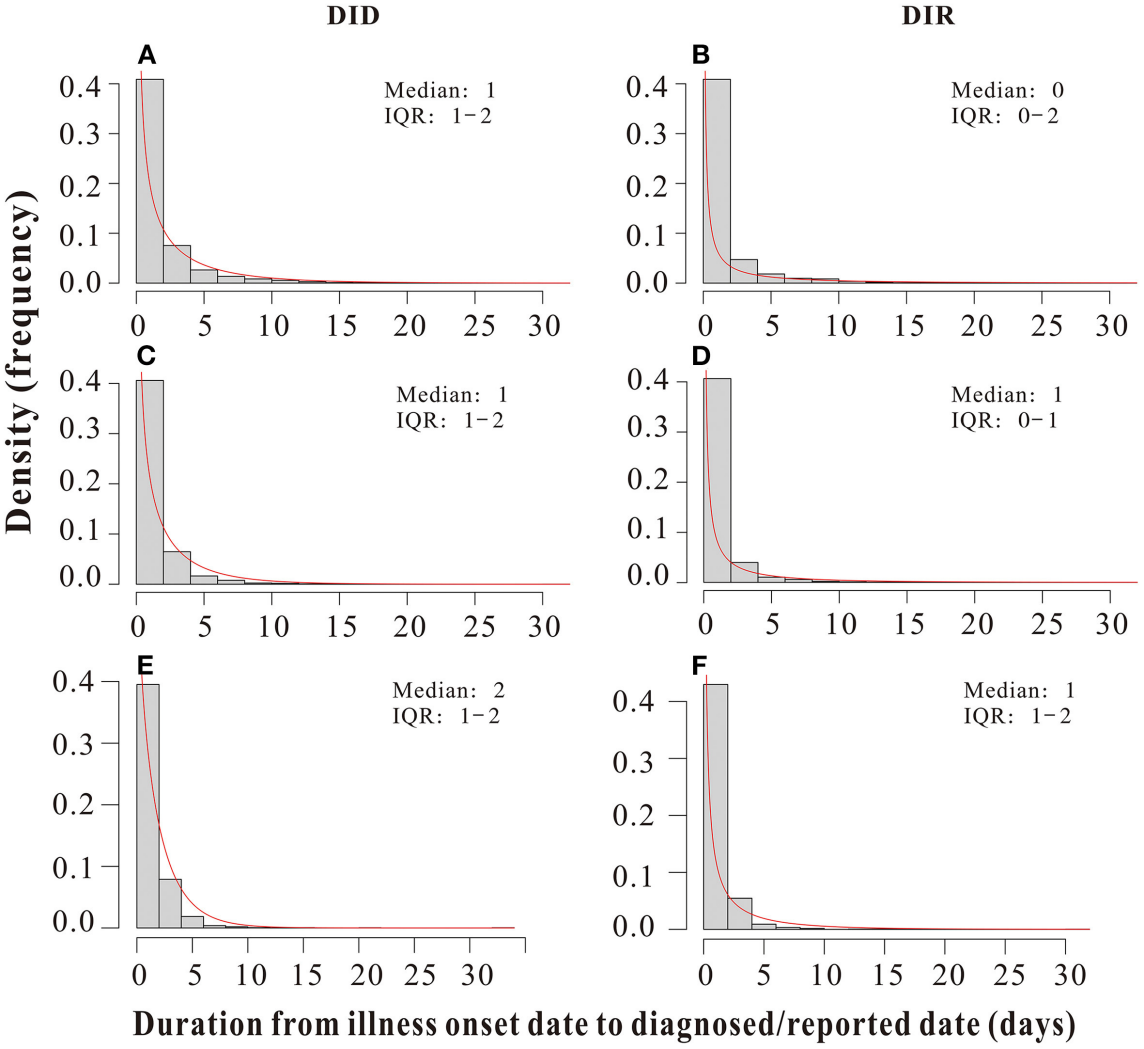
Duration from the illness onset date to the diagnosed date (*D*_*ID*_)/reported date (*D*_*IR*_) of all pre-Delta strains, Delta variant and Omicron variant outbreaks in China from March 28, 2020 to March 1, 2022. **(A,C,E)** *D*_*ID*_ of all pre-Delta strains, Delta variant and Omicron variant outbreaks, respectively. **(B,D,F)** *D*_*IR*_ of all pre-Delta strains, Delta variant and Omicron variant outbreaks, respectively.

The *D*_*ID*_ and *D*_*IR*_ of all outbreaks included in this study obey a gamma distribution, with durations basically concentrated in a range of 0–5 days. The median *D*_*ID*_of outbreaks due to pre-Delta strains, Delta variant and Omicron variant were all 1 day (inter-quartile range [IQR]: 1–2 days) (Figures 4A,C,E); The median *D*_*IR*_ of outbreaks of pre-Delta strains was 0 days (inter-quartile range [IQR]: 0–2 days); The median *D*_*IR*_ of Delta variant outbreaks was 1 day (inter-quartile range [IQR]: 0–1 days); The median *D*_*IR*_ of Omicron variant outbreaks was 1 day (inter-quartile range [IQR]: 1–2 days) (Figures 4B,D,F). The *D*_*ID*_ and *D*_*IR*_ of all outbreaks were in a range of 0–2 days, accounting for more than 75% (Table 2), which indicated that PHSMs applied were equally effective in case tracing and management. This result could also help to eliminate confounding factors that influenced the transmissibility of different variants.

### Comparison of duration and total number of cases between outbreaks of different strains

**Figure 5:**
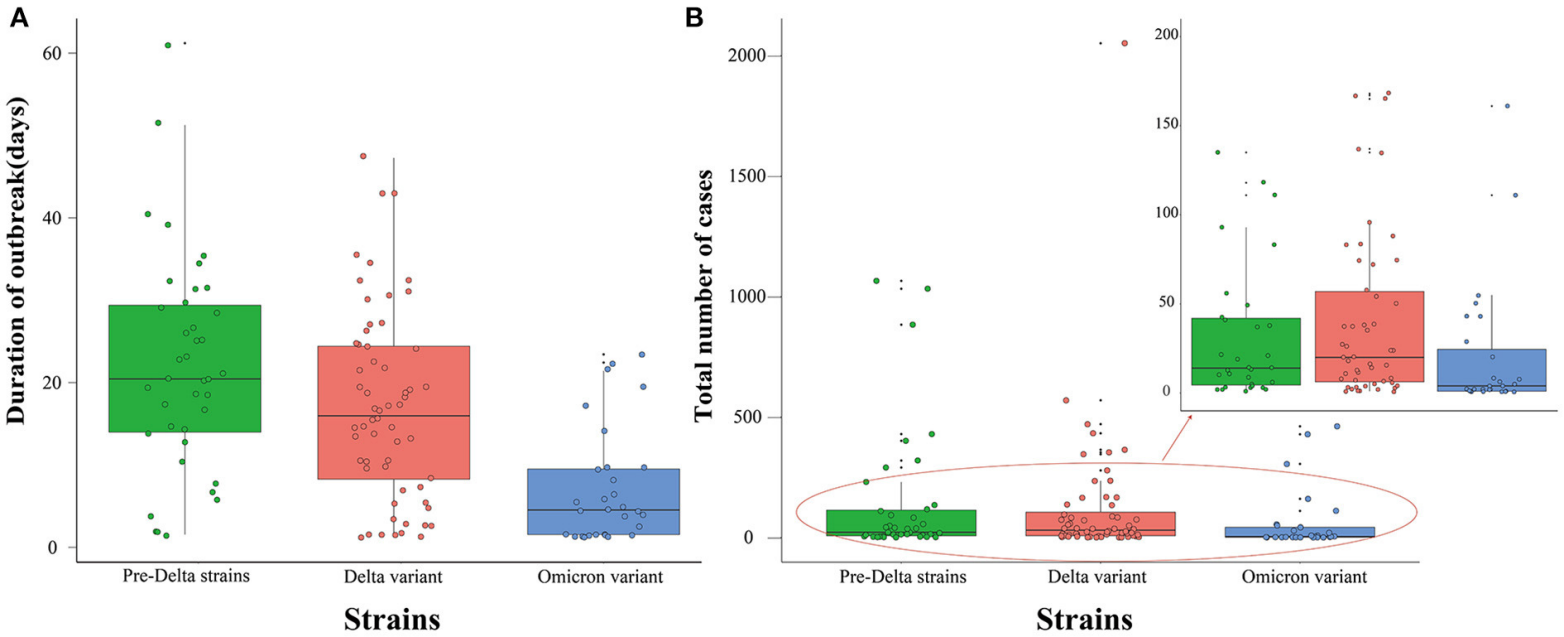
Duration of outbreak and total number of cases of all pre-Delta strains, Delta variant and Omicron variant outbreaks in China from March 28, 2020 to March 1, 2022. **(A)** Duration of outbreak. **(B)** Total number of cases.

Results showed that the duration of outbreaks of pre-Delta strains ranged from 1 to 61 days (median 20 days); duration of outbreaks of the Delta variant ranged from 1 to 47 days (median 16 days); and the duration of outbreaks of the Omicron variant ranged from 1 to 23 days (median 4 days). The range of the duration of outbreaks of pre-Delta strains was largest, and the differences between any two groups were statistically significant (*H* = 26.745, *P* < 0.05) (Figure 5A).

The total number of cases during outbreaks of pre-Delta strains ranged from 1 to 1,066 persons (median 21 persons); the total number of cases during Delta variant outbreaks ranged from 1 to 2,052 persons (median 31 persons); and the total number of cases during Omicron variant outbreaks ranged from 1 to 462 persons (median 5 persons). The range of the total number of cases during Omicron variant outbreaks was smallest, and the differences between any two groups were statistically significant (*H* = 9.236, *P* < 0.05) (Figure 5B).

## Discussion

This study is a large-scale sample size study with a long time span, we collected the data of all aggregated outbreaks in China since the first Wuhan-related wave. This also is the first comparative study in China to systematically review all local outbreaks in the country, and to analyze the differences in the characteristics and transmissibility of outbreaks involving different strains across multiple indicators using first-hand incidence data. The results will enable global scholars to deepen their understanding of Delta and Omicron variants, and will also provide theoretical references for the prevention and control of other possible future variants.

The WHO has reported several VOCs (Variants of Concern) (8, 33, 34). Since April 2021, the SARS-CoV-2 Delta variant has become the major variant worldwide, and the global prevalence of the Delta variant is also related to its enhanced interpersonal transmissibility (35). Global reports have documented rapid transmission characteristics of the Delta variant in different countries, such as Korea and France (16, 36). One study also found that the *R*_0_ of the Delta strain was 5.08 (37), which was similar to our results, our study showed that the median *R*_eff_ of Delta variants was 5.5. Currently the Omicron variant is the most prevalent strain in global outbreaks, and shows greater transmissibility than the Delta variant (38). The Omicron variant is estimated to be 100.3% more transmissible than the ancestral SARS-CoV-2 and 36.5% more transmissible than the Delta variant (8), A modeling study in South Africa showed that the mean *R*_eff_ of the Omicron strain was 7.57 (39), which was higher than our results, this may be related to the fact that this research was carried out during the early period of the Omicron outbreak in South Africa, which was also the early stage of the Omicron variant, and the transmissibility may be different from that of the later period. The *R*_eff_ value of Delta and Omicron outbreaks was >4, which indicates that the transmissibility of the Delta and Omicron variants has increased, resulting in more breakthrough infections. After a proportion of the population has received the vaccine, one individual can still infect more than 4 people, and the *R*_eff_ of the Omicron variant can be as high as 8 in this study, which suggests that the transmissibility of the strains increases as the virus mutates, a finding that is in accordance with the results of many studies (40–43).

Many studies have found that the average incubation period, average intergenerational interval, and average sequence interval of the Delta variant are significantly shorter than those of pre-Delta strains. The Delta variant is capable of spreading 4 generations within 10 days, with the fastest intergenerational transmission occurring in >24 h (24, 27). As for the Omicron variant, its estimated mean serial interval of 2.9 days was shorter than that observed for pre-Delta strains and the Delta variant, as found in studies conducted in South Korea (28, 44). The Omicron variant also has a growth advantage over the Delta variant due to its higher transmissibility, immunity evasion, and shorter serial interval (30). In this study, the epidemic curve and curve fitting results seen in outbreaks of the Delta and Omicron variants showed that there is a plateau stage for each epidemic curve, which indicates that Delta and Omicron outbreaks are more difficult to suppress than those of pre-Delta strains. After PHSMs were adopted, the disease continued to spread for about one incubation period. These findings provide us with a clearer and more precise picture of the transmissibility of different variants in the real world.

When both *R*_eff_ and *R*_t_ values are <1, the outbreak is less likely to spread continuously. We found that the values of *R*_eff_ and *R*_t_ fell below 1 following the rising stage of the outbreak. This decline in transmissibility may be related to many factors. Even though the vaccination rate in China is increasing, some studies have found that the vaccines have limited effect on the transmissibility of the virus. A UK community-based study suggests that vaccination alone is not sufficient to prevent all transmission of the Delta variant in the household setting, where exposure is close and prolonged (45). A cohort study also highlights that the effectiveness of vaccines in reducing transmission is minimal in the context of Delta variant circulation (46). Indeed, preclinical studies of adenovirus and mRNA candidate vaccines demonstrated persistent virus in nasal swabs despite preventing COVID-19. This suggests that systemically vaccinated patients, while asymptomatic, may still be become infected and transmit live virus from the upper airway (47).The scope and duration of outbreaks are largely influenced by PHSMs, and an outbreak can be controlled if the isolation scheme is implemented in time, and strict public health strategies are adopted (48). These results indicate that success in suppressing small-scale outbreaks can be mostly attributed to the PHSMs implemented by the Chinese government.

The discovery methods and timing of each outbreak are different, resulting in different entry points for controlling the spread of the epidemic. However, the outbreaks of Delta and Omicron strains have been successfully controlled after the platform period following an incubation period, which shows the effectiveness of our control measures. Our results also show that the *RDT* of the Delta and Omicron variants was higher than that of pre-Delta strains. The duration of Omicron outbreaks was significantly shorter than those of pre-Delta strains or the Delta variant in this study, since both pre-Delta and Delta strains have been superseded all around the world, Omicron is picking up the pace. We also found that the *D*_*ID*_ and *D*_*IR*_ of the Delta and Omicron variants were most often 0–2 days. This can be attributed to China's increasing experience in fighting the virus. Even in face of variants with increasing transmissibility, China continues to implement the “Dynamic Clearing” policy to suppress the spread of the outbreak. Comparisons between virus variants especially involving different outbreaks, should be taken with caution, since these outbreaks have a base-line differences in the diagnostic methods, which already differ according to the time of the outbreaks and the availability and accuracy of the technology (49). However, in our study, the DID and DIR of all the strains was most often 0–2 days, indicating that base-line differences in diagnostic methods of these outbreaks had no impact on the results.

The *R*_0_ value is usually used in public health research to measure the transmissibility of the disease. But in reality, the *R*_0_ value is not directly available due to the fact that not all individuals in the population are fully susceptible, and a percentage of the population has been vaccinated, in addition to the imposition of human intervention, the *R*_0_ value is therefore not directly available. Thus, the *R*_eff_ value is then generated to measure the disease transmissibility under interventions, and the *R*_t_ value is derived (50, 51). In predictions of the course of the COVID-19 epidemic, we usually judge the change in transmissibility at each stage of the outbreak by observing the rise and fall of the *R*_eff_ and *R*_t_ values, where the *R*_eff_ and *R*_t_ values = 1. At the key point when the *R*_eff_ and *R*_t_ values are <1, this indicates that transmission of the outbreak has been attenuated (51). However, there are situations in which time nodes with *R*_eff_ and *R*_t_ values <1 are different during the same outbreak, which implies that that there are some differences between *R*_eff_ and *R*_t_ values in measuring transmissibility. In describing the dynamics of the spread of the outbreak, the *R*_t_ value estimates the number of reproductions over time over a 5-day moving average, which represents the transmission trend of the outbreak over a period of time. However, we can usually use *R*_eff_ value to quantitatively express how many individuals one infected person can infect, which seems to have more explanatory and epidemiological value from a public health perspective. Some studies have pointed out that the *R*_t_ estimation may be biased due to certain complicating factors, including the use of inaccurate data sources in the calculation process and the use of the Serial Interval (SI) instead of the difficult-to-obtain Generation Time (GT) (52). In addition, *R*_t_ suffers from the shortcoming of averaging, which is an average of the total population and thus may mask the local variations. In this study, the difference in *R*_eff_ values between the pre-Delta strains, the Delta variant and the Omicron variant were statistically significant, whereas the difference in *R*_t_ between the Delta variant and pre-Delta strains was not statistically significant, and the median *R*_t_ values of Delta outbreaks and pre-Delta strains outbreaks were the same. This shows that *R*_eff_ is more likely to identify and measure the differences in transmissibility between different strains, and further indicates that *R*_eff_ is more suitable than *R*_t_ for assessing the transmissibility of the disease during an outbreak.

## Limitations

This study also has certain limitations. Firstly, in explaining the effectiveness of the PHSMs adopted in China, we have not undertaken an intensive analysis of the prevention and control effects of any specific intervention measure, such as closing schools and businesses, physical distancing etc. Secondly, the parameter values required to calculate *R*_eff_ and *R*_t_ were obtained by consulting reference literature, which may be slightly different from the actual parameters of each outbreak. In future research, we can consider using first-hand epidemiological survey data for more in-depth analysis. Finally, the vaccination rate, coverage rate and types of vaccine may affect the transmissibility of the strains, in the future research, if more detailed vaccine data can be obtained, we can then try to conduct more in-depth research to specifically analyze the extent of the impact of vaccines on transmissibility.

## Conclusion

The transmissibility of the Omicron variant is greater than that of pre-Delta strains or the Delta variant, and thus the Omicron variant is more difficult to control. At present, China's general policy of “Dynamic Clearing” has been extremely effective in dealing with the Omicron outbreak. There are certain differences between *R*_eff_ and *R*_t_ values in measuring transmissibility. The *R*_eff_ value is more suitable than the *R*_t_ value for evaluating the transmissibility of the disease in epidemic outbreaks.

## Data availability statement

The original contributions presented in the study are included in the article/Supplementary materials, further inquiries can be directed to the corresponding author.

## Author contributions

YN, LL, TC, and QL made substantial contributions to conception and design and critically revised the manuscript for important intellectual content. NY, XW, QL, and LL collected data. YN, LL, TC, SY, GA, BD, ZL, KL, TY, CL, ZeZ, and TC conceived the experiments. LL, WL, YW, ZhZ, BD, PL, JR, LL, SY, KL, and JH conducted experiments and analyzed results. YN, LL, TC, and QL wrote the manuscript. All authors have read and approved the final manuscript.

## Funding

This study was supported, in whole or in part, by the National Key R&D Program of China (2021ZD0113903) and (2021YFC2301604); Bill & Melinda Gates Foundation [INV-005832].

## Conflict of interest

The authors declare that the research was conducted in the absence of any commercial or financial relationships that could be construed as a potential conflict of interest.

## Publisher's note

All claims expressed in this article are solely those of the authors and do not necessarily represent those of their affiliated organizations, or those of the publisher, the editors and the reviewers. Any product that may be evaluated in this article, or claim that may be made by its manufacturer, is not guaranteed or endorsed by the publisher.

## Supplementary material

Supplementary Figure S1 Epidemic curve, *R*_eff_ value and *R*_t_ value of nine pre-Delta strains, **(A)** Results of curve fitting of the SEIR model with the onset data; **(B)** The time-dependent reproduction number (*R*_t_) of the ongoing COVID-19 outbreak.

Supplementary Figure S2 Epidemic curve, *R*_eff_ value and *R*_t_ value of 13 Delta variant outbreaks, **(A)** Results of curve fitting of the SEIR model with the onset data; **(B)** The time-dependent reproduction number (*R*_t_) of the ongoing COVID-19 outbreak.

Supplementary Figure S3 Epidemic curve, *R*_eff_ value and *R*_t_ value of eight Omicron variant outbreaks, **(A)** Results of curve fitting of the SEIR model with the onset data; **(B)** The time-dependent reproduction number (*R*_t_) of the ongoing COVID-19 outbreak.

Supplementary Table S1 The value of *R*^2^ of nine pre-Delta strains, 13 Delta variant and eight Omicron variant outbreaks.
